# Ultra-processed foods: a concept in need of revision to avoid targeting healthful and sustainable plant-based foods

**DOI:** 10.1017/S0007114523000430

**Published:** 2023-10-28

**Authors:** Mark J. Messina, John L. Sievenpiper, Patricia Williamson, Jessica Kiel, John W. Erdman

**Affiliations:** 1 Soy Nutrition Institute Global, Washington, DC, USA; 2 Department of Nutritional Sciences and Medicine, Temerty Faculty of Medicine, University of Toronto; Division of Endocrinology & Metabolism, Department of Medicine, St. Michael’s Hospital; Li Ka Shing Knowledge Institute, St. Michael’s Hospital, Toronto, ON, Canada; 3 Scientific and Regulatory Affairs, Research and Development, Cargill, Minneapolis, MN, USA; 4 Scientific and Clinical Affairs, Baltimore, MD, USA; 5 Department of Food Science and Human Nutrition, Division of Nutritional Sciences and Beckman Institute, University of Illinois at Urbana/Champaign, Urbana, IL, USA

We read with interest the invited commentary by Dr Mark Lawrence supporting the utility of the NOVA food classification system^(1)^. However, we take issue with his perspective on our recently published article in which we make two fundamental points^(2)^. First, the common criticisms of ultra-processed foods (UPF) do not apply to soya-based meat and dairy alternatives more so than they do to their animal-based counterparts, meat and cows’ milk, despite the former being classified as UPF and the latter as unprocessed/minimally processed foods. Second, NOVA is overly simplistic and does not adequately evaluate the nutritional attributes of meat and dairy alternatives based on soya. Simply put, soya burgers are not Twinkies, even though NOVA similarly classifies these products. Rather than focusing on the crux of our argument, Dr Lawrence notes the association of several authors with the soyafood industry, that is, classic ad hominem reasoning. Dr Lawrence also criticises us for not considering the ‘… broader public health, environmental and social implications of such innovations (e.g., soy burgers) relative to food processing innovations to promote existing non-UPF nutritious plant-source protein foods such as minimally processed legumes and nuts’. We fully support greater consumption of legumes and nuts and efforts to promote their intake. However, the products in question are designed to replace meat and dairy products not legumes and nuts. Therefore, the critical comparisons are between hamburgers and soya burgers and cows’ milk and soyamilk. We therefore standby our opinion that NOVA does a disservice to the public by suggesting that because soya burgers and soyamilk are NOVA-classified as UPF, they should be avoided. These foods can aid in the transition to and maintenance of plant-based diets.
